# Risk factors for poor engagement with a smart pillbox adherence intervention among persons on tuberculosis treatment in Ethiopia

**DOI:** 10.1186/s12889-023-16905-z

**Published:** 2023-10-14

**Authors:** Amare Worku Tadesse, Martina Cusinato, Gedion Teferra Weldemichael, Tofik Abdurhman, Demelash Assefa, Hiwot Yazew, Demekech Gadissa, Amanuel Shiferaw, Mahilet Belachew, Mamush Sahile, Job van Rest, Ahmed Bedru, Nicola Foster, Degu Jerene, Katherine Linda Fielding

**Affiliations:** 1 TB Centre, Department of Infectious Disease Epidemiology, London, School of Hygiene & Tropical Medicine (LSHTM) , London, UK; 2 Bristol Medical School, Population Health Sciences, Bristol, UK; 3 KNCV Tuberculosis Plus, The Hague, Ethiopia; 4 KNCV Tuberculosis Plus, The Hague, Netherlands; 5 School of Public Health, University of the Witwatersrand, Johannesburg, South Africa

**Keywords:** Drug-sensitive tuberculosis, Smart pillbox, Digital adherence technologies, Poor engagement, Ethiopia

## Abstract

**Background:**

Non-adherence to tuberculosis treatment increases the risk of poor treatment outcomes. Digital adherence technologies (DATs), including the smart pillbox (EvriMED), aim to improve treatment adherence and are being widely evaluated. As part of the Adherence Support Coalition to End TB (ASCENT) project we analysed data from a cluster-randomised trial of DATs and differentiated care in Ethiopia to examine individual-factors for poor engagement with the smart pillbox.

**Methods:**

Data were obtained from a cohort of trial participants with drug-sensitive tuberculosis (DS-TB) whose treatment started between 1 December 2020 and 1 May 2022, and who were using the smart pillbox. Poor engagement with the pillbox was defined as (i) > 20% days with no digital confirmation and (ii) the count of days with no digital confirmation, and calculated over a two evaluation periods (56-days and 168-days). Logistic random effects regression was used to model > 20% days with no digital confirmation and negative binomial random effects regression to model counts of days with no digital confirmation, both accounting for clustering of individuals at the facility-level.

**Results:**

Among 1262 participants, 10.8% (133/1262) over 56-days and 15.8% (200/1262) over 168-days had > 20% days with no digital confirmation. The odds of poor engagement was less among participants in the higher stratum of socio-economic position (SEP) over 56-days. Overall, 4,689/67,315 expected doses over 56-days and 18,042/199,133 expected doses over 168-days were not digitally confirmed. Compared to participants in the poorest SEP stratum, participants in the wealthiest stratum had lower rates of days not digitally confirmed over 168-days (adjusted rate ratio [RR_a_]:0.79; 95% confidence interval [CI]: 0.65, 0.96). In both evaluation periods (56-days and 168-days), HIV-positive status (RR_a_:1.29; 95%CI: 1.02, 1.63 and RR_a_:1.28; 95%CI: 1.07, 1.53), single/living independent (RR_a_:1.31; 95%CI: 1.03, 1.67 and RR_a_:1.38; 95%CI: 1.16, 1.64) and separated/widowed (RR_a_:1.40; 95%CI: 1.04, 1.90 and RR_a_:1.26; 95%CI: 1.00, 1.58) had higher rates of counts of days with no digital confirmation.

**Conclusion:**

Poorest SEP stratum, HIV-positive status, single/living independent and separated/ widowed were associated with poor engagement with smart pillbox among people with DS-TB in Ethiopia. Differentiated care for these sub-groups may reduce risk of non-adherence to TB treatment.

## Background

Non-adherence to treatment remains to be one of the challenges that tuberculosis programmes face. It can result in increased risk of poor treatment outcomes, development of drug resistant strains, transmission of TB in the community, increased morbidity and mortality [1].

The literature has documented several factors that are known to affect treatment adherence. These include individual factors such as age, sex, treatment phase, HIV co-infection, stigma, low income, lower education level, lack of support; provider factors such as health care workers’ skills, training, and attitudes; and health system factors including patient load and health care staffing [2–10]. Digital adherence technologies (DATs), such as the smart pillbox, short message service (SMS) or video supported therapy, aim to improve treatment adherence, ideally resulting in better treatment outcomes and reducing treatment recurrence [11]. These interventions are based on patient engagement with the technology, such as a pillbox opening, considered a proxy for treatment adherence. Real-time monitoring of DAT engagement by health care workers allows additional support to be offered to patients who appear to have issues with treatment adherence [11].

The Adherence Support Coalition to End TB (ASCENT) project is conducting cluster-randomised trials (CRTs) in five countries with varied epidemiology of TB and HIV, to evaluate the effectiveness of DATs with differentiated care in improving treatment outcomes [12, 13]. In this context, understanding engagement with the DAT with respect to individual-level and facility-level factors is of interest in order to understand intervention delivery. Furthermore, in resource limited settings, identifying factors that contribute to poor engagement with DAT could facilitate optimal implementation of adherence interventions and promote patient-centred differentiated care to improve TB treatment adherence. Hence, good engagement with the DAT is important for showing potential effectiveness of these interventions.

Ethiopia is among the 30 high TB burden countries in the world with an estimated TB incidence rate of 143 per 100,000 population and mortality rate of 18 per 100,000 population [14]. Studies from Ethiopia have variable and inconsistent findings regarding non-adherence to treatment for tuberculosis ranging between 9 and 36% [5, 10, 15, 16]. Early detection of non-adherence as measured through poor engagement with the DAT and information on contributing factors are needed to understand how interventions, including smart pill boxes, can improve adherence through person-centred care.

This analysis examined the individual-factors associated with poor engagement with the DAT among adults with pulmonary drug-sensitive TB (DS-TB) using the smart pillbox participating in the ASCENT project in Ethiopia. Our findings will inform a pragmatic approach of measuring treatment adherence using smart pillbox to identify and support people with TB at risk of poor medication adherence.

## Methods

### Study design/setting

The study area is in Addis Ababa city and Oromia region in Ethiopia. The ASCENT trial is a pragmatic three-arm cluster randomised trial (CRT), with health facility as the unit of randomisation. Facilities were randomised, in a ratio of 1:1:1, to either a DAT intervention (smart pillbox or medication labels) with daily monitoring of adherence and differentiated care, or standard of care. Adults (≥ 18 years) with pulmonary DS-TB initiating treatment in the health facilities participating in the trial were offered enrolment. Following informed consent, participants received the intervention allocated to the facility they enrolled from (Trial registration: PACTR202008776694999). Details of the trial design are described elsewhere [12]. Those in the medication labels arm facilities who did not have access to a phone were offered a pillbox.

Participants using the smart pillbox, received the box with dosing instruction and anti-TB drugs were placed were inside. Based on the participants’ preference, the boxes were configured with an audio-visual alert to remind patients to take their medication at a pre-defined time. Each time participants opened the box to take their medication, their “daily dose” was automatically logged on to the Everwell Hub web-based platform (https://www.everwell.org) via a built-in mobile internet connection. For days where the box was not opened, the platform flagged a missed daily dose. This platform allowed TB care providers to access and evaluate real-time dosing data of each participant and offer differentiated care, including SMS reminders, phone calls and home visits, as appropriate.

### Study population

The study population for this analysis was nested within an ongoing pragmatic CRT of smart pillbox and medications labels, and included adult patients, who started treatment for DS-TB between 1 December 2020 and 1 May 2022, initiating on the pillbox within 14 days of treatment start. Participants with completely missing baseline or dosing data were excluded, as were those switching to MDR-TB treatment, missing treatment outcomes, or whose diagnosis changed to not TB. Engagement with technology was based on digital logging of the box being opened, over the first 56 and 168 days of treatment, representing the intensive phase of treatment and full course of treatment for TB, respectively.

### Measures

To investigate the effect of risk factors on poor engagement with the technology among adults with DS-TB, individual-level factors known to affect adherence to treatment for tuberculosis were assessed. These factors were collected at study enrolment by interview or abstracted from the TB register and categorised as socio-demographic or clinical factors. The socio-demographic factors were sex, age, education, marital status, and wealth index. The clinical factors were HIV status, previous TB status and type of TB (whether the TB was diagnosed bacteriologically or not). A wealth index was created representing household socioeconomic position (SEP) using principal component analysis [17, 18]. Using a polychoric analysis of household assets (livestock, land, and house ownership), characteristics (cooking fuel type, toilet/sewage facilities, source of drinking water, number of rooms per person, frequency of income), and consumer goods (bicycle, truck, cart, motorcycle, bed, refrigerator, lamp, mattress, mitad, mobile, ratio, sofa, television), we constructed a dual-component wealth index. This allows an additional dimension of wealth, expressed by the second component, which helps to limit the introduction of urban bias through a better representation of rural patterns of wealth [19]. This index was subsequently grouped into quintiles, measuring household SEP. A facility-level (health-system) measure of the percentage of poor treatment outcome (death, lost to follow-up or treatment failure) among adults with DS-TB, evaluated in a cohort prior the start of the trial, was calculated. This percentage was grouped into terciles based on the distribution at the cluster-level to account for baseline health facility-level characteristics.

The main outcome for this study was poor engagement with smart pillbox and measured using two approaches: (i) a binary response defined as > 20% days with no digital confirmation and (ii) the count of days with no digital confirmation. Both outcomes were measured over the two time periods of 56-days (intensive phase) and 168-days (full course of 6 month TB treatment). For the binary outcome the denominator was defined as the number of days from the start date of the DAT to the earliest of (i) treatment stop or (ii) analysis time period (day 56 and 168 from treatment start). Treatment stop was either at the time of (i) transfer to another facility, completion of treatment, on-treatment death, lost to follow-up or treatment failure, or (ii) participant withdrawal from the intervention either due to the participant wanting to stop or technical issues with the device. The count outcome was measured as the number of days with no recorded digital pillbox opening. Digital confirmation on a treatment-day refers to the participant opening the pillbox on the day, and is assumed to be a proxy for a dose taken.

### Statistical analysis

Descriptive statistics, including proportions, standard deviations and ranges, were computed to describe age and sex distributions, HIV prevalence and poor engagement with the smart pillbox. Logistic random effects regression was used to model the binary outcome of at least > 20% days with no digital confirmation. Negative binomial random effects regression was used to model the count of days with no digital confirmation, and the denominator, as described previously, used as an offset. Both models accounted for clustering of individuals at the facility-level. Risk factors investigated included age, sex, previous TB, bacteriological confirmation, HIV status, level of education, marital status, and SEP. Changes in the magnitude of estimates between crude and (age and sex) adjusted measures of effect were evaluated for each potential risk factor. Following age- and sex-adjustment three variables were identified to investigate further in multivariable analysis, based on confidence intervals excluding one for either outcome or time period. Separate, fully-adjusted models, were constructed for these variables where adjustment was guided by a conceptual framework, avoiding adjustment for intermediate variables. All available data based on our inclusion criteria for this study were used in the analysis and no formal sample size calculation was conducted. Precision of estimates were judged by reviewing the 95% confidence intervals. To avoid issues of data sparsity for the binary outcome model adjustment was restricted to a minimum of 10 outcomes per variable [20, 21]. Complete case analyses were conducted as the percentage of the total sample size with missing data was less than 2.5%. Data analysis was conducted using Stata 17.

Written informed consent was obtained from all participants in the ASCENT trial. The trial has approval from London School of Hygiene & Tropical Medicine Ethics Committee (19,120), United Kingdom; WHO Ethical Review Committee (ERC.0003297), Switzerland; Addis Ababa City Administration Health Bureau Public Emergency and Health Research Directorate Institutional Review Board (AA16238/227), and Oromia Regional Health Bureau Public Emergency and Health Research Directorate Institutional Review Board, Ethiopia (BEFO/HBTFH/1–16/10415).

## Results

**Fig. 1:**
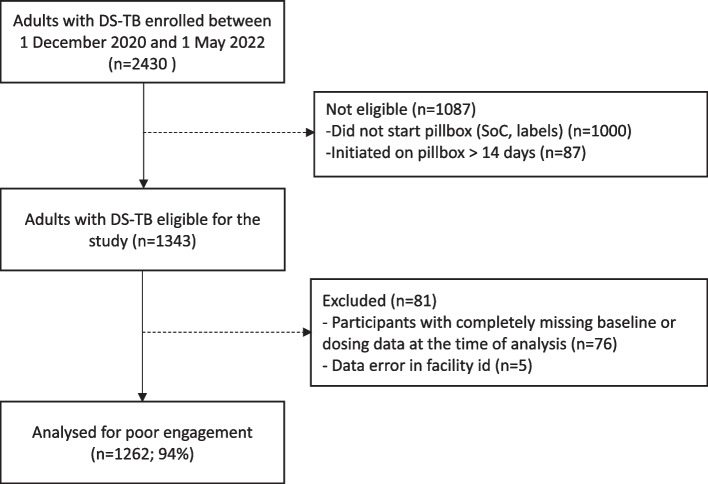
Flow diagram for study participants

Between 1 December 2020–1 May 2022, 2,430 adults with DS-TB were enrolled across the three arms of the parent study, excluding those who had switched to an MDR regimen, or diagnosis changed to not TB or TB outcome unknown. A total of 1,000 participants were in the standard of care arm or started medication labels, and a further 87 started the pillbox > 14 days after treatment start, leaving 1,343 participants. Of these, 76 had no available baseline or DAT engagement data and five had other data errors that could not be resolved. This resulted in a total of 1262 participants (94%) included in this analysis (Fig. 1). These participants were enrolled from 43 facilities; 26 facilities randomised to the pillbox arm and 17 randomised to the medication labels arm, for whom patients with no mobile phone access used the pillbox. The mean number of participants per facility was 29.3 (standard deviation [SD] 27.3) with a range of 1 to 135. The mean age was 33.9 years (SD 13.8, range 18 to 99), 59.2% (747/1262) were male and 40.8% (515/1262) were female, and prevalence of HIV was 12.5% (157/1257). Half (50.9%, 643/1262) of the participants had no or less than primary level education and 66.5% (839/1261) had bacteriologically confirmed TB (See Table 1).

**Table 1:** Socio-demographic and clinical characteristics of study participants (*N* = 1262)

	n	%
**Age in years**		
18 to 24	364	28.8
25 to 34	401	31.8
35 +	497	39.4
**Sex**		
Female	515	40.8
Male	747	59.2
**HIV status**^a^		
Negative	1100	87.5
Positive	157	12.5
**Previous TB**		
No	1183	93.8
Yes	78	6.2
**Education**		
None	183	14.5
Less than primary	460	36.5
Primary completed	220	17.4
Secondary or more	399	31.6
**Marital status**		
Single	324	25.7
Single/independent	234	18.5
Married/cohabiting	573	45.4
Separated/widowed	131	10.4
**SEP**		
Poorest	248	20.0
Second	247	20.0
Middle	247	20.0
Fourth	247	20.0
Wealthiest	247	20.0
**BC confirmed**		
Yes	839	66.5
No	422	33.5

^a^HIV status at baseline. *TB* Tuberculosis, *SEP* socio-economic position *BC* bacteriological confirmation. There were 5 patients with unknown HIV status, 1 patient with unknown TB history, 26 with missing data on SEP and 1 patient with missing bacteriological confirmation

Over the intensive phase and full course of TB treatment, 10.5% (133/1262) and 15.8% (200/1262) of participants had > 20% of days with no digital confirmation, respectively. Table 2 displays the crude odds ratio [OR] (and 95% confidence interval [CI]) of poor engagement with technology for the risk factors explored, as well as the corresponding age-and-sex-adjusted ORs (and 95%CI). Participants in the fourth stratum of SEP had a 51% lower (95%CI 6% to 75%) odds of poor engagement with technology over intensive phase – compared to those in the poorest stratum; and that participants in the wealthiest stratum of SEP had a 41% (95%CI 0% to 65%) lower odds of poor engagement with technology over full course of TB treatment – compared to those in the poorest stratum. Over intensive phase, being clinically diagnosed was associated with a 55% increase in odds of poor engagement, though this effect was not observed over full course of TB treatment.

**Table 2:** Poor engagement with technology defined as proportion of participants with > 20% days with no digital confirmation over intensive and full course of TB treatment

	**POOR ENGAGEMENT (> 20% DAYS WITH NO DIGITAL CONFIRMATION) OVER INTENSIVE PHASE**						**POOR ENGAGEMENT (> 20% DAYS WITH NO DIGITAL CONFIRMATION) OVER FULL COURSE**					
	10.5%	(133/1262)	ORc	ORc 95%CI	ORa	ORa 95%CI	15.8%	(200/1262)	ORc	ORc 95%CI	ORa	ORa 95%CI
**% poor outcome**^ƚ^												
< 4%	10.0%	(51/510)	1	1	1	1	15.9%	(81/510)	1	1	1	1
> 4% to 7%	13.1%	(50/381)	1.33	(0.70, 2.55)	1.34	(0.70, 2.57)	15.7%	(60/381)	0.98	(0.53, 1.82)	1.00	(0.53, 1.87)
> 7%	8.6%	(32/371)	0.78	(0.40, 1.52)	0.78	(0.40, 1.53)	15.9%	(59/371	0.97	(0.54, 1.77)	0.99	(0.54, 1.82)
**Age**, in years												
18 to 24	11.5%	(42/364)	1	1	1	1	17.9%	(65/364)	1	1	1	1
25 to 34	8.5%	(34/401)	0.69	(0.42, 1.12)	0.70	(0.43, 1.14)	14.7%	(59/401)	0.76	(0.51, 1.12)	0.74	(0.50, 1.11)
35 +	11.5%	(57/497)	0.94	(0.61, 1.45)	0.97	(0.63, 1.51)	15.3%	(76/497)	0.78	(0.54, 1.14)	0.77	(0.53, 1.12)
**Sex**												
Female	11.8%	(61/515)	1	1	1	1	14.4%	(74/515)	1	1	1	1
Male	9.6%	(72/747)	0.76	(0.52, 1.10)	0.77	(0.53, 1.11)	16.9%	(126/747)	1.18	(0.86, 1.63)	1.21	(0.88, 1.67)
**HIV status**^§^												
Negative	10.2%	(112/1100)	1	1	1	1	15.9%	(175/1100)	1	1	1	1
Positive	12.7%	(20/157)	1.26	(0.74, 2.12)	1.24	(0.72, 2.13)	15.9%	(25/157)	0.98	(0.61, 1.57)	1.06	(0.65, 1.73)
**Previous TB**												
No	10.6%	(125/1183)	1	1	1	1	16.0%	(189/1183)	1	1	1	1
Yes	10.3%	(8/78)	1.09	(0.50, 2.37)	1.08	(0.50, 2.36)	14.1%	(11/78)	0.96	(0.49, 1.88)	0.97	(0.49, 1.91)
**Education**												
None	8.2%	(15/183)	1	1	1	1	14.8%	(27/183)	1	1	1	1
Less than primary	11.5%	(53/460)	1.50	(0.81, 2.78)	1.72	(0.91, 3.24)	17.6%	(81/460)	1.28	(0.79, 2.08)	1.17	(0.70, 1.93)
Primary completed	10.9%	(24/220)	1.45	(0.72, 2.90)	1.74	(0.84, 3.61)	15.5%	(34/220)	1.16	(0.66, 2.05)	1.02	(0.56, 1.85)
Secondary or more	10.3%	(41/399)	1.35	(0.71, 2.56)	1.58	(0.80, 3.09)	14.5%	(58/399)	1.05	(0.63, 1.75)	0.93	(0.54, 1.60)
**Marital status**												
Single	10.8%	(35/324)	1	1	1	1	14.5%	(47/324)	1	1	1	1
Single/independent	9.4%	(22/234)	0.86	(0.48, 1.53)	0.87	(0.49, 1.56)	17.9%	(42/234)	1.30	(0.81, 2.07)	1.35	(0.84, 2.16)
Married/cohabiting	10.8%	(62/573)	0.94	(0.60, 1.49)	0.96	(0.59, 1.56)	14.8%	(85/573)	0.97	(0.65, 1.44)	1.11	(0.73, 1.71)
Separated/widowed	10.7%	(14/131)	0.90	(0.46, 1.77)	0.81	(0.39, 1.68)	19.8%	(26/131)	1.29	(0.75, 2.23)	1.59	(0.88, 2.89)
**SEP**												
Poorest	12.5%	(31/248)	1	1	1	1	19.0%	(47/248)	1	1	1	1
Second	7.7%	(19/247)	0.59	(0.31, 1.09)	0.61	(0.33, 1.14)	16.2%	(40/247)	0.82	(0.51, 1.34)	0.81	(0.50, 1.32)
Middle	14.6%	(36/247)	1.16	(0.67, 2.00)	1.20	(0.69, 2.07)	17.4%	(43/247)	0.87	(0.53, 1.40)	0.86	(0.53, 1.39)
Fourth	6.5%	(16/247)	0.49	(0.25, 0.94)	0.49	(0.25, 0.95)	13.8%	(34/247)	0.68	(0.41, 1.14)	0.68	(0.41, 1.13)
Wealthiest	10.9%	(27/247)	0.84	(0.47, 1.52)	0.85	(0.47, 1.53)	12.1%	(30/247)	0.59	(0.35, 1.00)	0.59	(0.35, 1.01)
**BC confirmed**												
Yes	9.4%	(79/839)	1	1	1	1	15.7%	(132/839)	1	1	1	1
No	12.8%	(54/422)	1.56	(1.06, 2.29)	1.55	(1.05, 2.28)	16.1%	(68/422)	1.11	(0.79, 1.54)	1.11	(0.80, 1.55)

Crude (ORc) and adjusted (ORa) odds ratio and 95% confidence intervals (CI) calculated using logistic regression with random effects. Adjusted models condition on age and sex only

^ƚ^Facility level percentage with poor outcomes in the previous year (§) HIV status at baseline. *TB* Tuberculosis, *SEP* socio-economic position, *BC* bacteriological confirmation. There were 5 patients with unknown HIV status, 1 patient with unknown TB history, 26 with missing data on SEP and 1 patient with missing bacteriological confirmation

When poor engagement was measured using counts of days with no digital confirmation, a total of 4,686 (6.9%) doses were not digitally confirmed (out of 67,315 doses required) over the intensive phase of treatment and 18,042 (9.0%) were not digitally confirmed (out of 199,133 required) over the full course of TB treatment (Table 3). Participants in the wealthiest stratum of SEP had a 21% (95%CI 4% to 35%) reduction in the rate of days not digitally confirmed over full course of TB treatment compared to the poorest stratum, after adjusting for age and sex. No such pattern was observed over intensive phase. HIV-positive participants had a 29% higher rate of no digital confirmation over intensive phase (95%CI 2% to 63%) and a 28% higher rate of doses missed over full course of TB treatment (95%CI 7% to 53%) compared to HIV negative participants, after adjusting for age and sex. There was also some evidence of higher rates of no digital confirmation among those single/ living independent and those separated/widowed compared to single participants living with parents. The age-and-sex adjusted rate ratio (RR) for those single/living independent was 1.31 (95%CI 1.03 to 1.67) over intensive phase and 1.38 (95%CI 1.16 to 1.64) over full course of TB treatment; for those separated/widowed the corresponding RR were 1.40 (95%CI 1.04 to 1.90) and 1.26 (95%CI 1.00 to 1.58).

**Table 3:** Poor engagement with technology defined as proportion of participants with counts of days with no digital confirmation over intensive and full course of TB treatment

	**POOR ENGAGEMENT (COUNTS OF DAYS WITH NO DIGITAL CONFIRMATION) OVER INTENSIVE PHASE**						**POOR ENGAGEMENT (COUNTS OF DAYS WITH NO DIGITAL CONFIRMATION) OVER FULL COURSE**					
	Λ = 0.070	(4686/67315)	RRc	RRc 95%CI	RRa	RRa 95%CI	Λ = 0.091	(18,042/199133)	RRc	RRc 95%CI	RRa	RRa 95%CI
**% -poor outcome**												
< 4%	0.067	(1820/27307)	1	–	1	–	0.093	(7574/81424)	1	–	1	–
> 4% to 7%	0.084	(1704/20370)	1.01	(0.88, 1.27)	1.02	(0.81, 1.28)	0.085	(5040/59171)	0.85	(0.71, 1.02)	0.86	(0.72, 1.02)
> 7%^a^	0.059	(1162/19638)	1.10	(0.87, 1.38)	1.12	(0.89, 1.41)	0.093	(5428/58508)	0.94	(0.79, 1.12)	0.96	(0.81, 1.15)
**Age, in years**												
18 to 24	0.080	(1543/19256)	1	–	1	–	0.100	(5701/56987)	1	–	1	–
25 to 34	0.058	(1249/21570)	0.89	(0.73, 1.09)	0.89	(0.73, 1.09)	0.088	(5683/64224)	0.86	(0.74, 1.00)	0.86	(0.74, 0.99)
35 +	0.072	(1894/26489)	0.87	(0.72, 1.05)	0.87	(0.72, 1.05)	0.085	(6658/77892)	0.81	(0.71, 0.93)	0.81	(0.70, 0.93)
**Sex**												
Female	0.071	(1952/27322)	1	–	1	–	0.084	(6699/80183)	1	–	1	–
Male	0.068	(2734/39993)	0.96	(0.82, 1.12)	0.97	(0.82, 1.13)	0.095	(11,343/118920)	1.06	(0.94, 1.20)	1.08	(0.96, 1.21)
**HIV status**^§^												
Negative	0.067	(3955/58796)	1	–	1	–	0.090	(15,686/174676)	1	–	1	–
Positive	0.086	(712/8278)	1.22	(0.97, 1.53)	1.29	(1.02, 1.63)	0.098	(2329/23738)	1.17	(0.99, 1.39)	1.28	(1.07, 1.53)
**Previous TB**												
No	0.070	(4420/63124)	1	–	1	–	0.092	(17,118/186625)	1	–	1	–
Yes	0.064	(265/4168)	1.16	(0.85, 1.59)	1.18	(0.86, 1.62)	0.074	(923/12455)	1.06	(0.84, 1.34)	1.09	(0.86, 1.38)
**Education**												
None	0.060	(590/9765)	1	–	1	–	0.089	(2570/28867)	1	–	1	–
Less than primary	0.075	(1832/24495)	1.12	(0.88, 1.42)	1.10	(0.85, 1.41)	0.098	(6975/71451)	1.19	(1.00, 1.43)	1.11	(0.92, 1.35)
Primary completed	0.067	(789/11774)	1.03	(0.77, 1.36)	1.01	(0.75, 1.36)	0.081	(2833/35115)	1.06	(0.86, 1.30)	0.97	(0.77, 1.20)
Secondary or more	0.069	(1475/21281)	1.09	(0.85, 1.40)	1.05	(0.81, 1.38)	0.089	(5664/63670)	1.10	(0.92, 1.33)	1.01	(0.83, 1.23)
**Marital status**												
Single	0.065	(1124/17321)	1	–	1	–	0.084	(4367/51838)	1	–	1	–
Single/independent	0.069	(871/12552)	1.28	(1.01, 1.62)	1.31	(1.03, 1.67)	0.099	(3641/36595)	1.34	(1.13, 1.59)	1.38	(1.16, 1.64)
Married/cohabiting	0.072	(2206/30546)	1.13	(0.92, 1.38)	1.22	(0.98, 1.50)	0.088	(7918/90404)	1.03	(0.89, 1.19)	1.13	(0.97, 1.32)
Separated/widowed	0.070	(485/6896)	1.27	(0.96, 1.68)	1.40	(1.04, 1.90)	0.104	(2116/20266)	1.10	(0.89, 1.37)	1.26	(1.00, 1.58)
**SEP**												
Poorest	0.085	(1136/13328)	1	–	1	–	0.109	(4305/39618)	1	–	1	–
Second	0.053	(695/13167)	0.86	(0.66, 1.11)	0.86	(0.66, 1.11)	0.088	(3421/38888)	0.91	(0.76, 1.10)	0.91	(0.75, 1.09)
Middle	0.094	(1231/13076)	1.12	(0.88, 1.44)	1.12	(0.88, 1.43)	0.105	(4014/38276)	0.98	(0.82, 1.18)	0.97	(0.81, 1.17)
Fourth	0.052	(693/13357)	0.90	(0.69, 1.16)	0.89	(0.69, 1.15)	0.081	(3246/40311)	0.88	(0.73, 1.05)	0.86	(0.72, 1.04)
Wealthiest	0.061	(794/13091)	0.86	(0.66, 1.11)	0.87	(0.67, 1.12)	0.069	(2655/38259)	0.79	(0.65, 0.95)	0.79	(0.65, 0.96)
**BC confirmed**												
Yes	0.065	(2936/45052)	1	–	1	–	0.090	(12,081/133772)	1	–	1	–
No	0.079	(1749/22240)	1.00	(0.84, 1.18)	1.00	(0.84, 1.18)	0.091	(5960/65308)	1.01	(0.89, 1.14)	1.01	(0.89, 1.14)

Crude (RRc) and adjusted (RRa) rate ratio and 95%(CI) calculated using negative binomial regression with random effects. Adjusted models condition on age and sex only

(Λ) rate calculated as counts of no digital confirmation over total treatment-days. ^a^Facility level proportion of poor outcomes in the previous year (§) HIV status at baseline. *TB* Tuberculosis, *BC* bacteriological confirmation, *SEP* socio-economic position. There were 5 patients with unknown HIV status, 1 patient with unknown TB history, 26 with missing data on SEP and 1 patient with missing bacteriological confirmation

Table 4 displays the results of the multivariable models assessing HIV status, SEP and marital status as risk factors for poor engagement with technology, using the two outcome measures. After adjusting for age, sex, previous TB, level of education, marital status and SEP, HIV-positive participants had 27% higher rates of no digital confirmation over the intensive phase (95%CI 0% to 62%) compared to HIV negative participants and 29% higher rates (95%CI 7% to 55%) when the outcome was assessed over full course of TB treatment. The association was not observed for the binary poor engagement outcome over intensive phase or full course of TB treatment. For SEP, after adjusting for age, sex, previous TB, level of education and marital status, the wealthiest stratum had 18% lower rates of no digital confirmation over full course of TB treatment (95%CI 0% to 34%), with similar patterns for the binary outcome, albeit with confidence intervals including one. In the analysis of count of no digital confirmation over full course of TB treatment, being single/living independent, and separated/widowed showed higher rates of poor engagement. The facility-level measure showed no association with either poor engagement outcome.

**Table 4:** Results of the multivariable models assessing HIV status and SEP as risk factors for poor engagement with technology using > 20% days and counts of days with no digital confirmation over intensive phase and full course of TB treatment

	** > 20% DAYS WITH NO DIGITAL CONFIRMATION**				**COUNTS OF DAYS WITH NO DIGITAL CONFIRMATION**			
	**INTENSIVE PHASE**		**FULL COURSE**		**INTENSIVE PHASE**		**FULL COURSE**	
	ORa	ORa 95%CI	ORa	ORa 95%CI	RRa	RRa 95%CI	RRa	RRa 95%CI
**HIV status**								
Negative	1	–	1	–	1	–	1	–
Positive	1.26	(0.72, 2.21)	0.97	(0.58, 1.61)	1.27	(0.99, 1.62)	1.29	(1.07, 1.55)
**SEP**								
Poorest	1	–	1	–	1	–	1	–
Second	0.58	(0.31, 1.08)	0.80	(0.49, 1.31)	0.85	(0.65, 1.10)	0.9	(0.75, 1.08)
Middle	1.10	(0.62, 1.92)	0.88	(0.53, 1.43)	1.09	(0.85, 1.40)	0.98	(0.81, 1.18)
Fourth	0.44	(0.22, 0.87)	0.71	(0.42, 1.19)	0.88	(0.67, 1.15)	0.88	(0.73, 1.07)
Wealthiest	0.73	(0.38, 1.40)	0.62	(0.35, 1.10)	0.85	(0.64, 1.12)	0.82	(0.66, 1.00)
**Marital status**								
Single	1	–	1	–	1	–	1	–
Single/independent	0.77	(0.41, 1.43)	1.22	(0.74, 2.01)	1.23	(0.96, 1.59)	1.34	(1.12, 1.60)
Married/cohabiting	0.86	(0.52, 1.42)	1.04	(0.67, 1.61)	1.17	(0.94, 1.46)	1.10	(0.94, 1.29)
Separated/widowed	0.74	(0.35, 1.57)	1.48	(0.80, 2.72)	1.35	(0.99, 1.85)	1.26	(0.99, 1.59)

Adjusted (ORa) odds ratio and 95% confidence intervals (CI) calculated using logistic regression with random effects. Adjusted (RRa) rate ratio and 95%(CI) calculated using negative binomial regression with random effects. Model for HIV status at baseline (*N* = 1231, DF = 16) adjusted for age, sex, previous TB, level of education, marital status, SEP, and facility. Model for SEP (*N* = 1235 DF = 15) adjusted for age, sex, previous TB, level of education, marital status, and facility. Model for marital status (*N* = 1235 DF = 15) adjusted for age, sex, previous TB, level of education, SEP, and facility. The variable facility represents proportion of poor outcomes per facility in the previous year. SEP socio-economic position

## Discussion

This study analysed individual and facility factors associated with poor engagement with the smart pillbox in a population of 1262 adults with DS-TB receiving treatment in a predominantly urban setting in Ethiopia. Over the intensive phase of treatment, 7.0% of doses were not digitally confirmed and 10.5% of participants had > 20% of treatment days with no digital confirmation. These became, 9.1% and 15.8% respectively when the outcome was evaluated over full course of TB treatment. We found some evidence that higher relative SEP was associated with better engagement with technology particularly over full course of treatment for TB. Rates of no digital confirmation were higher in HIV positive compared to HIV negative participants, over the intensive phase and full course of treatment for TB. Finally, those single/ living independent or separated/widowed had higher poor engagement for both outcomes, particularly over full course of treatment for TB. Rather than showing different factors for intensive versus the full course of treatment period, our results were generally consistent across the two time periods.

Engagement with the smart pillbox over the entire 6-month treatment period has been reported in recent trials, with results comparable to our observations. In a cluster-randomised trial from China with every other day dosing, in the pillbox arm, 13.9% of treatment days were not digitally confirmed and 17% of participants had at least 20% days with no digital confirmation [22]. In the TB Mate study from South Africa, among participants in the pillbox arm, 11.5% of days were not digitally confirmed and 18.5% of participants had at least 20% of treatment days without digital confirmation [23].

Comparison of our results to the literature on factors associated with poor engagement is limited to what is known about factors associated with more direct measures of adherence, as little is reported on DAT engagement. The relationship between lower SEP and increased risk of general poor health has been well explored in the literature [24–26]. People with low SEP, and particularly many people with TB, are burdened with more complex life issues, including lack of access to nutritious food, lack of access to health care, be overworked or stressed and less privacy at home due to overcrowding [5, 10, 27–31]. This may make them prioritise basic life needs rather than their overall health and/ or treatment care, be less conversant with TB treatment and ultimately lead to poor engagement to the technology that was provided to support their TB treatment.

In our study, participants with TB/HIV co-infection were more likely to miss doses of their anti-TB treatment medication, based on the proxy of poor engagement with the DAT, measured by counts of days with no digital confirmation. This finding was consistent with previous studies conducted in Sub Saharan African countries, including Ethiopia. Pill burden, adverse effects of anti-TB medication and fear of stigma among people with TB/HIV co-infection have been documented to be more common compared to people with TB only [6, 27, 32–34]. Furthermore, less motivation to take medication among people with TB/HIV co-infection has been reported in studies conducted in Sub-Saharan Africa [35, 36]. These stressors may likely exacerbate forgetfulness, psychological distress, and non-disclosure of HIV status to family, which in turn could negatively influence their medication adherence and engagement to DAT. Therefore, our study may highlight the relevance of social relationships in improving adherence to treatment, especially in resource limited settings.

We found participants who were single/ living independent and separated/ widowed had higher likelihood of missing doses as evidenced by poor engagement with the DAT, after adjustment for age and other factors. This could reflect lower social capital in this subgroup. Although we did not capture a more direct measure of social capital defined as psychosocial assets which shape health, it includes the support from household- or community members that individuals with TB receive in their treatment [17]. Social factors for non-adherence such as stigma and limited knowledge of TB may have a pronounced detrimental effect in the livelihood of these sub-groups and deprive them of social network that could have addressed the social barriers to TB treatment adherence. Previous studies have shown that providing social support that could involve financial incentives, emotional support, and informational provision from one’s social network, can improve adherence to TB treatment [37–41]. Another pathway for those single could be through having to travel longer periods for work and not taking pillbox due to the inconvenience [5].

A facility-level measure of percentage of people with TB with poor treatment outcomes prior to our study, aiming to reflect facility and individual-level engagement with care, was not found to be associated with participant engagement with the DAT. More detailed data on facility-level measures such facility infrastructure, staff leadership, engagement with the interventions, and patient–provider relationship would be required to have a better understanding of the role of facility-level factors in DAT engagement [42].

Our study aimed to understand engagement with the DAT among people with TB. Comparing studies evaluating treatment adherence is often challenging without a gold standard comparator test, such as urine isoniazid testing. Some studies have suggested good sensitivity and/ or specificity but others have shown poor agreement, particularly with a SMS-based DAT. Our study included participants from 43 health facilities in two regions in Ethiopia, though mainly limited to more urban areas. We used two measures of DAT poor engagement, measured over two time periods—a binary variable reflecting > 20% of treatment days with no digital confirmation, aligning with commonly used categorisation of adherence in the literature [22, 43–47] and a count of treatment days with no digital confirmation. The binary measure is likely to have resulted in reduced power to detect differences by subgroups. We constructed a measurement of SEP using two principal components, rather than the first one, as proposed by Martel et al. (2021). This allows incorporation of the additional dimension of wealth thereby limiting the introduction of urban bias through a better representation of rural patterns of wealth. Measures of stigma and several dimensions social capital which may influence DAT engagement were not collected as part of the study. Our study sample is representative of the trial population and missing data were minimal. Results from the two outcome approaches are broadly similar, reflecting consistency of baseline effects on poor engagement. Despite careful adjustment for confounders in the regression models guided by conceptual frameworks, one limitation of the study is we cannot discount residual confounding due to unmeasured confounders.

## Conclusion

Participants’ poor engagement with smart pillbox and rates of days with no digital confirmation increased over TB treatment time. Poorest SEP stratum, HIV-positive status, single/ living independent and separated/ widowed were individual-level factors associated with poor engagement with the DAT among adults with DS-TB receiving treatment in Ethiopia. Additional support, such as counselling, psychosocial and peer support to these sub-groups is recommended to reduce risk of non-adherence to TB treatment. Provider and facility level factors associated with poor engagement during the treatment course warrants further study.

## Data Availability

The datasets generated and analysed during the current study are not publicly available due to pending publication of main trial results which were used under license for the current study but available from the corresponding author on reasonable request.

## Abbreviations

ASCENT: Adherence Support Coalition to End TB
CI: Confidence interval
CRT: Cluster randomised trial
DAT: Digital adherence technology
DS-TB: Drug-sensitive tuberculosis
HIV: Human Immunodeficiency Virus
MDR-TB: Multidrug resistant tuberculosis
OR_c_: Crude odds ratio
OR_a_: Adjusted odds ratio
RR_c_: Crude rate ratio
RR_a_: Adjusted rate ratio
SEP: Socioeconomic position
SMS: Short message service
TB: Tuberculosis
WHO: World Health Organisation
